# Gene expression profiling reveals underlying molecular mechanism of hepatoprotective effect of *Phyllanthus niruri* on thioacetamide-induced hepatotoxicity in Sprague Dawley rats

**DOI:** 10.1186/1472-6882-13-160

**Published:** 2013-07-05

**Authors:** Zahra A Amin, Mohammed A Alshawsh, Mustafa Kassim, Hapipah M Ali, Mahmood A Abdulla

**Affiliations:** 1 Department of Pharmacognosy, College of Pharmacy, Hawler Medical University, Erbil 44001, Iraq; 2 Department of Pharmacology, Faculty of Medicine, University of Malaya, Kuala Lumpur 50603, Malaysia; 3 Department of Anesthesiology, Faculty of Medicine, University of Malaya, Kuala Lumpur 50603, Malaysia; 4 Department of Chemistry, Faculty of Science, University of Malaya, Kuala Lumpur 50603, Malaysia; 5 Department of Biomedical Science, Faculty of Medicine, Kuala Lumpur 50603, Malaysia

**Keywords:** *Phyllanthus niruri*, Hepatoprotective, Gene expression, Active constituents

## Abstract

**Background:**

The liver plays an essential role in the body by regulating several important metabolic functions. Liver injury is associated with the distortion of these functions causing many health problems. Pharmaceutical drugs treat liver disorders but cause further damage to it. Hence, herbal drugs are used worldwide and are becoming increasingly popular.

**Methods:**

The hepatoprotective activity of *Phyllanthus niruri* (PN) was evaluated against liver cirrhosis induced by thioacetamide (TAA) in male Sprague Dawley rats. Rats received intraperitoneal injections of thioacetamide (TAA, 200 mg/kg, b.w. three times weekly) for eight weeks. Daily treatments with plant extract (200 mg/kg) were administered orally for eight weeks. At the end of the study, hepatic damage was evaluated by monitoring transforming growth factor (TGFβ), collagen α1 (Collα1), matrix metalloproteinase-2 (MMP2) and tissue inhibitor of matrix metalloproteinase-1 (TIMP1) gene expression by real-time PCR. Moreover, different chromatographic techniques including column chromatography, thin layer chromatography, and Ultra Performance Liquid Chromatography (UPLC) with Liquid Chromatography/Mass Spectrometry (LC/MS) were used to isolate the active constituents of the plant.

**Results:**

The results revealed that treatment with PN significantly reduced the effect of thioacetamide toxicity and exhibited effective hepatoprotective activity. The mechanism of the hepatoprotective effect of PN is proposed to be by normalizing ROSs. Additionally, PN treatment regulated the expression of TGFβ, Collα1, MMP2, and TIMP1 genes. In the active fraction of *P. niruri*, the isolated chemical constituents were 4-O-caffeoylquinic acid and quercetin 3-O-rhamnoside.

**Conclusions:**

The results of the present study indicate that PN ethanol extracts possess hepatoprotective activity that is most likely because of the isolated chemical constituents.

## Background

*Phyllanthus niruri* has been used in folk medicine as an antipyretic, analgesic, or anti-inflammatory treatment, and treatment of other symptoms suggests antihistamine effects. Moreover, the decoction of the whole plant has been used orally against diarrhea and topically to treat jaundice. Crushed leaves together with leaves of *Eupatorium odoratum* and lime are applied on boils [1]. Previous studies have revealed the therapeutic potential of *Phyllanthus niruri* to treat genitourinary infections, venereal diseases, and kidney or bladder stones. Moreover, *P. niruri* is reported to act as a urinary inhibitor of calcium oxalate crystallization and an effective treatment for urolithiasis by interfering in the growth and aggregation of calcium oxalate crystals [2-4]. The reported anti-hyperuricemic action might be because of its uricosuric activity through an xanthine oxidase inhibitory effect [5].

Many reports in the literature have verified the protective activity of *Phyllanthus niruri* against various drug- and toxin-induced hepatic disorders. Earlier studies [6] have shown that extracts of *P. niruri* have demonstrated hepatoprotective activity against the carbon tetrachloride induced lipid peroxidation in the livers of rats, which was determined by raised serum enzyme levels. Although the effects of aqueous extracts of *P. niruri* against carbon tetrachloride (CCL_4_)-induced liver, kidney and testes injuries have been studied [7], Manjrekar *et al.* concluded that the hepatoprotective and antioxidant activity of this plant was associated with adverse effects on kidney and testes. In the study by Bhattacharjee *et al*. [8], the hepatoprotective potential of the protein isolated from *P. niruri* against CCL_4_-induced liver damage was investigated. These results suggested that this protein protected the liver against oxidative stress and stimulated liver repair mechanisms. Additionally, Harish *et al*. [6] investigated the antioxidant activity of extracts of *P. niruri* against CCL_4_-induced liver damage. They demonstrated that membrane lipid peroxidation (LPO) inhibition was confirmable by pre-treatment with the extracts.

In our previous research, we proved that *P. niruri* possesses hepatoprotective activity against thioacetamide-induced liver cirrhosis. Acute toxicity was studied, and the results demonstrated that *P. niruri* extract was non toxic when applied to SD rats. Significant differences were observed between thioacetamide-treated rats (200 mg/kg) and high or low dose (200 mg/kg and 100 mg/kg) *P. niruri-*treated rats in the body and liver weights, total antioxidant capacity, liver biochemical parameters, oxidative stress enzyme and lipid peroxidation levels. Moreover, *P. niruri* treatment effectively restored the histological and morphological observations closer to their normal appearances [9].

The goal of this study was to study the mechanism that induces the hepatoprotective activity of *Phyllanthus niruri* ethanol extract in protecting liver cirrhosis induced by thioacetamide in Sprague Dawley rats by monitoring the expression of transforming growth factor beta (TGFβ1), tissue inhibitors of metalloproteinases (TIMP1), matrix metalloproteinase (MMP2), and collagen alpha (Collα1) gene expression by real-time PCR. Moreover, the active constituents of the *Phyllanthus niruri* were isolated by separating the crude extract into several fractions using flash column chromatography and thin layer chromatography. Subsequently, the immunomodulatory activity for all fractions was tested to examine their abilities to proliferate human peripheral blood mononuclear cells (PBMCs). LC/MS was performed on the fraction that exhibited higher proliferation activity on the PBMCs.

## Methods

### Preparation of plant extract

*Phyllanthus niruri* plant was gained from Ethno Resources Sdn Bhd, identified and a voucher specimen (voucher number KLU46618) was kept. By the method of Zahra *et al.*[10], a crude ethanol extract was prepared by drenching 100 g of it in 1000 mL of 95% ethanol (1:10 w/v) for 72 hours at 25°C. The mixture was filtered and distilled under reduced pressure at 45°C by a rotary evaporator. The crude extract was maintained at −20°C until further experiments were done.

### Chemicals and apparatus

In brief, 95% (v/v) ethanol, filter paper (Whatman No. 1), Thioacetamide, xylazine, ketamine, formalin, hematoxylin, and eosin were purchased from Sigma-Aldrich (Gillingham, UK). RNA*later* solution (Applied Biosystems, Foster City, CA, USA), QIAamp RNA blood mini kit (Qiagen, Germantown, MD, USA), RNase-free DNase set (Qiagen), agarose gels, Tris-borate-EDTA (10× TBE) (Applied Biosystems), ethidium bromide, loading dye (Promega, Madison, WI, USA) and a UV gel documentation system (Vilber Lourmat, Fisher Scientific Sdn Bhd). High Capacity RNA-to-cDNA Master Mix, TaqMan Fast Advanced Master Mix, ultrapure DNase-free water (Applied Biosystems) were used to perform the reverse transcription and real-time PCR. Transforming growth factor beta (TGFβ1), tissue inhibitors of metalloproteinases (TIMP1), matrix metalloproteinase (MMP2), collagen alpha (Collα1), hypoxanthine phosphoribosyltransferase 1 (Hprt1), and peptidylprolyl isomerase A (Ppia) were the genes of interest.

Silica gel 60 powder (0.063–0.200 mm, 70–230 mesh), silica gel F254 plates (20 × 20 cm, 0.2 mm), HPLC grade n-hexane, HPLC grade ethyl acetate, HPLC grade methanol, HPLC grade acetonitrile were purchased from (Merck, Germany), a Kontes column (2 × 30 cm) with an EYEL-4 pump (Rikakikai, Tokyo, Japan) and a Milli-Q water purification system (Millipore, Billerica, MA, USA) were used to perform the HPLC analysis.

### Experimental design

The animal protocol was agreed by the Ethics Committee for Animal Experimentation, Medicine Faculty, University Malaya, Kuala Lumpur, Malaysia, under Ethic number PM/28/08/2010/MAA (R). The animals were cared for according to the “Guide for the Care and Use of Laboratory Animals”, published by the National Academy of Science. The animals were provided with water and standard pellet *ad libitum*.

Sprague Dawley rats (24 male) weighing 190–260 grams were divided into 3 groups randomly of 8 rats each. The experimental groups were as follows: healthy controls (vehicle control group), thioacetamide controls (liver cirrhosis control), and the *P. niruri* treatment group. The plant extract was suspended in Tween 20 (10%) and administered by oral gavage (5 mL/kg body weight). Thioacetamide was suspended in dH_2_O (2 mL/kg body weight) and injected intraperitoneally to the rats. **Group no**. **1** (normal group) treated daily with Tween 20 orally and injected with dH_2_O three times weekly intraperitoneally (ip) for two months. **Group no**. **2** (TAA group) treated daily with Tween 20 and injected with TAA (200 mg/kg) three times weekly for two months. The above mentioned procedure was following the method of Alshawsh *et al*. [11]. **Group no**. **3** (PN-treated group) treated daily with the PN extract (200 mg/kg) orally and injected with TAA (200 mg/kg) three times weekly for two months.

### Sample collection

After two months, each rat was fasted for 24 hours prior to sacrificing. A perfusion was performed under ketamine and xylazine (1:10 v/v) anesthesia, and rats were quickly sacrificed by exsanguination of the jugular vein. A small portion of the livers were kept immediately in an RNA*later* solution (Applied Biosystems) for gene expression analysis and kept at −80°C until the purification experiment was performed. The gene expression assays were performed using the following TaqMan gene expression workflow: RNA isolation and purification, RNA transcription RNA to cDNA, and amplification of cDNA and target genes by real-time PCR. The gene assays used in this study were transforming growth factor beta (TGFβ1), tissue inhibitors of metalloproteinases (TIMP1), matrix metalloproteinase (MMP2), and collagen alpha (Collα1), and hypoxanthine phosphoribosyltransferase 1 (Hprt1) and peptidylprolyl isomerase A (Ppia) were used as housekeeping genes.

### Gene expression profile

#### RNA isolation and purification

RNA was extracted from the frozen liver using a QIAamp RNA Blood Mini Kit following the manufacturer’s protocol. Briefly, 30 mg of frozen RNAlater-stabilized liver tissue was weighed immediately (without allowing the tissue to thaw), then disrupted and grinded by using a mortar and pestle. QIAshredder spin columns were used for homogenization and separation of the tissue lysates. The column digestion of DNA was performed during RNA purification using an RNase-free DNase set according to the manufacturer’s instructions. Finally, total RNA was stored at −70°C until further use.

RNA purity was quantified using a spectrophotometer using a 10 mm quartz cuvette. With a 40× dilution factor, absorbance was measured at 230, 260, 280, and 320 nm, and the following ratios were calculated: 260/280 and 260/320.

RNA integrity was measured by agarose gels electrophoresis. Electrophoresis buffer tris-borate-EDTA (10× TBA) was prepared to fill the electrophoresis tank and cast the gel. A 0.5% (w/v) solution of agarose in electrophoresis buffer was prepared. Ethidium bromide was added to the molten gel to a final concentration (0.5 μg/mL) and then mixed thoroughly by gentle swirling. A small-toothed comb (allows 1 μL sample/well) was positioned on the plate to form complete wells. The 60°C agarose solution was poured into the mold and allowed to set at room temperature for 30–45 minutes. The gel was mounted into the tank, and the electrophoresis buffer was added to cover the gel at a depth of 1 mm.

RNA samples (1 μL each) were loaded after mixing with the loading dye. Subsequently, the RNAs were allowed to migrate toward the positive anode. The gel ran for 30 minutes at 95 V until the migrated distance was 75% through the gel. The gels were examined under UV light to observe the discrete 18S and 28S ribosomal RNA bands.

#### Reverse transcription of RNA to cDNA

cDNA was synthesized using High Capacity RNA-to-cDNA Master Mix in a reaction plate according to the manufacturer’s instructions.

#### Amplification of cDNA by real-time PCR

Real-time PCR was performed using a StepOnePlus System and TaqMan Fast Advanced Master Mix (Applied Biosystems). The total reaction volume (10 μL) consisted of the following: 1 μL cDNA (20 ng), 5 μL TaqMan Fast Advanced Master Mix, 0.5 μL of each TaqMan Gene Expression Assay, and 3.5 μL ultrapure DNase-free water. The cycle parameters were as follows: UNG incubation at 50°C for 2 min, polymerase activation at 95°C for 20 s, denaturation at 95°C for 1 s and then annealing and extension at 60°C for 20 s.

#### Real-time PCR data normalization

The real-time PCR work was done in triplicate for each sample. Two endogenous control genes, hypoxanthine phosphoribosyltransferase 1 (Hprt1) and peptidylprolyl isomerase A (Ppia), were used for normalization. The comparative CT method was used to calculate to calculate the relative amount of the transcripts in all groups, and genes were normalized to the endogenous controls. The final value was normalized to the Hprt1 and Ppia genes and qualified to the normal control values of the investigated genes. The formula is as follows:

$$\Delta \Delta \mathrm{CT}=\Delta \mathrm{CT}\left(\mathrm{sample}\right)-\Delta \mathrm{CT}\left(\mathrm{normal}\right)$$

Where ∆CT is the difference in CT between the targeted gene and housekeeping controls by minimizing the average CT of the controls. The fold-change calculated as: **2**^-∆∆**CT**^.

### Chromatography profile

#### Flash column chromatography (FCC)

Plant fractionations were performed following the method of Fraga *et al*. [12]. Flash column chromatography was performed on silica gel 60 (0.063–0.200 mm, 70–230 mesh) from (Merck, Germany) using a Kontes column (2 × 30 cm) with an EYEL-4 pump (Rikakikai, Tokyo, Japan). The elution process to extract plant fractions (1 g/5 mL methanol) began with the most non-polar solvent (n-hexane), and then a continuous gradient elution (n-hexane – ethyl acetate – methanol – acetonitrile) was applied that concluded with the most polar solvent (dH_2_O), which was purified by a Milli-Q water purification system (Millipore).

#### Thin layer chromatography (TLC)

The obtained fractions were dissolved in methanol at 10 mg/mL to perform thin layer chromatography (TLC) with silica gel F254 (20 × 20 cm, 0.2 mm) plates. The analyses were achieved in the following: n-hexane – ethyl acetate, ethyl acetate – methanol, methanol – acetonitrile, and acetonitrile – water.

#### Ultra Performance Liquid Chromatography (UPLC) and Liquid Chromatography/Mass Spectrometry (LC/MS)

A Waters Synapt HDMS system in TOF-mode was used to perform Ultra Performance Liquid Chromatography (UPLC) and HDMS-mode was used to perform mass spectrometry that was equipped with an ACQUITY PDA Detector and ACQUITY UPLC BEH C_18_ column (1.7 μm, 2.1 × 50 mm).

The flow rate was 0.5 mL/min, and the injection volume was 3 μL. The analyses were performed using binary gradients of Milli-Q water (with 0.1% formic acid) (solvent A) and HPLC grade acetonitrile (with 0.1% formic acid) (solvent B) with the following elution profile: from 0 min: 0% (B) in (A); 7 min: 100% (B) in (A); 10 min: 100% (B) in (A); 11 min: 0% (B) in (A).

#### Statistical analysis and data management

Real-time PCR data were analyzed using GenEx program. (GenEx software, http://www.multid.se); fold changes were calculated, and *T*-test was used to examine the differences between groups for all genes. “The Dictionary of Natural Products on DVD” software (CRC Press, Taylor and Francis Group, http://www.netbeans.org) was used to analyze the chromatography profiling data.

## Results

### Integrity of RNA

**Figure 1:**
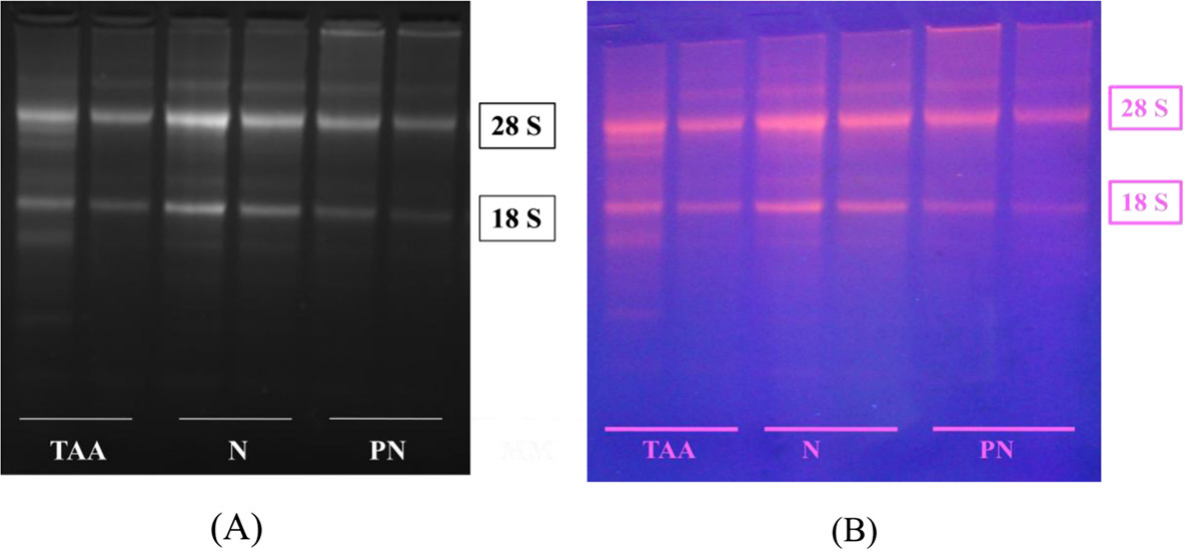
A typical ethidium bromide-stained agarose gel shows the integrity of the extracted RNA; (A) visualized under a Vilber Lourmat gel documentation system (B) visualized under UV light.

Total RNA was extracted from the liver tissues, and the quantity of RNA was determined by reading the absorbance at 260 nm spectrophotometrically with an ND-2000 NanoDrop Spectrophotometer (Thermo Fisher Scientific, Wilmington, DE, USA). The ratio of the absorbance readings at 260 nm and 280 nm was used to indicate the quality of RNA. The 260/280 ratio for our RNA preparation ranged from 1.6–2.1; these values suggested good quality RNA. The integrity of RNA was checked by agarose gel electrophoresis. Discrete 28S and 18S ribosomal RNA bands were obtained in each case. The 28S rRNA band was approximately twice as large as the 18S rRNA band, indicating that the extracted RNA was intact and could be used in RT-PCR. Figure 1 shows a typical ethidium bromide-stained agarose RNA gel.

### Real-time PCR analysis

**Figure 2:**
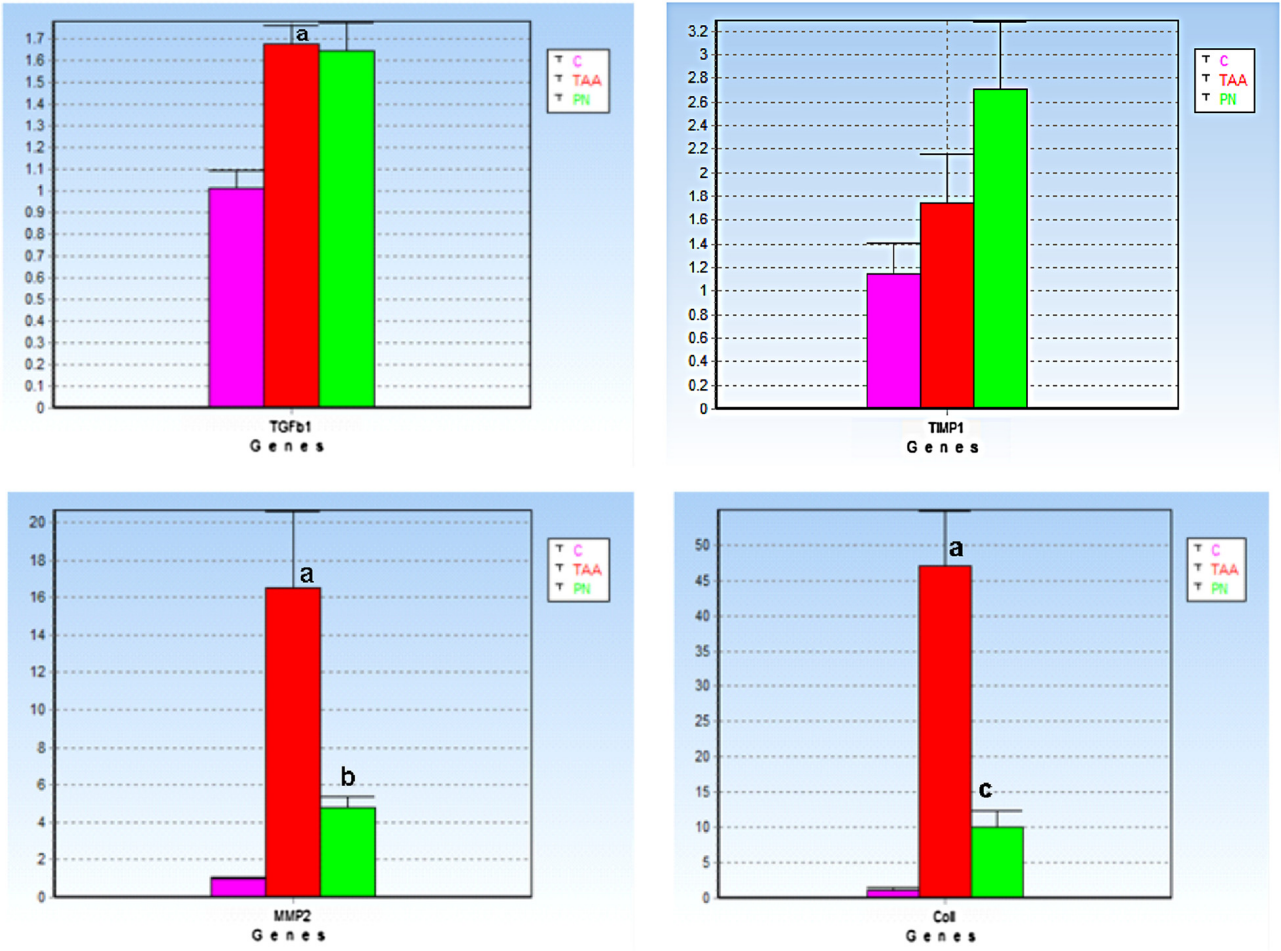
**Real-time PCR analysis shows the relative fold changes of transforming growth factor beta (TGFβ), matrix metalloproteinase 2 (MMP2), tissue inhibitors of metalloproteinases 1 (TIMP1), and collagen alpha (Collα1) between all experimental groups.** Values expressed as the mean ± S.E.M. Different superscripts state significant differences. **(a)** Indicates significance versus the Normal group at *P* ≤ 0.05; **(b)** indicates significance versus the TAA-treated group at *P* ≤ 0.05 and **(c)** indicates significance versus the TAA-treated group at *P* ≤ 0.01.

Ct values of real-time PCR data were calculated using GenEX software and normalized to the reference genes HPRT1 and Ppia. The analysis showed significant differences in mRNA expression levels of the investigated genes between the controls and TAA- and PN-treated rats. In the control rats, the mRNA levels of TGFβ, collα1, MMP2 or TIMP1 were unchanged suggesting that the hepatic satellite cells (HSCs) were in their quiescent state (Figure 2). In the TAA-treated group, hepatic expression levels of all investigated genes were upregulated. The upregulation was significant at (*P* < 0.01) in the TAA-treated group compared with the PN-treated group for the genes TGFβ (1.677 ± 0.120), Collα1 (47.062 ± 7.716) and MMP2 (14.500 ± 3.528). However, the difference was non-significant for TIMP1 (1.738 ± 0.486).

Oral administration of PN before cirrhosis induction prevented and resolved the activation of HSCs, and the remaining cells expressed decreased levels of TGFβ, Collα1, and MMP2 compared with the TAA-treated group as shown in Figure 2.

### Chromatography profile

**Figure 3:**
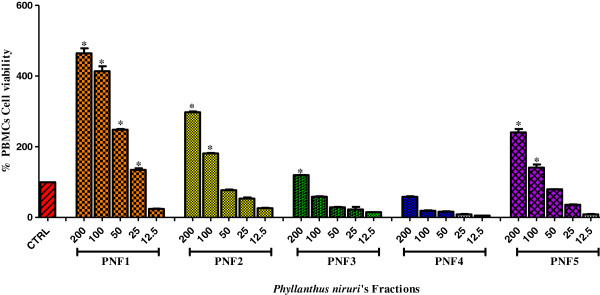
**The effects of *P. niruri *fractions on human peripheral blood mononuclear cell (PBMC) proliferation.** Data are expressed as the mean ± SEM for triplicates; (*) indicates significance versus the control group (CTRL = dH_2_O) at *P* ≤ 0.05.

After crude extraction of *P. niruri,* the ethanol extract was objected to flash column chromatography to separate the constituents of the extracts according to molecular size, molecular mass, and polarity. Therefore, 12 fractions were obtained, and by performing thin layer chromatography, the subsequent fractions with the same retention factor and spot colors after visualizing under UV light at 240 nm and 360 nm were combined to yield five fractions (PNF1, PNF2, PNF3, PNF4, and PNF5). The best resolutions of plates were given by acetonitrile-water. Subsequently, the immunomodulatory activity for all fractions was tested to examine their abilities to proliferate human peripheral mononuclear cells (PBMCs). As shown in Figure 3, PN fractions showed high activities as a percent of viability to proliferate PMBCs; the fraction with the highest activity was PNF1.

**Figure 4:**
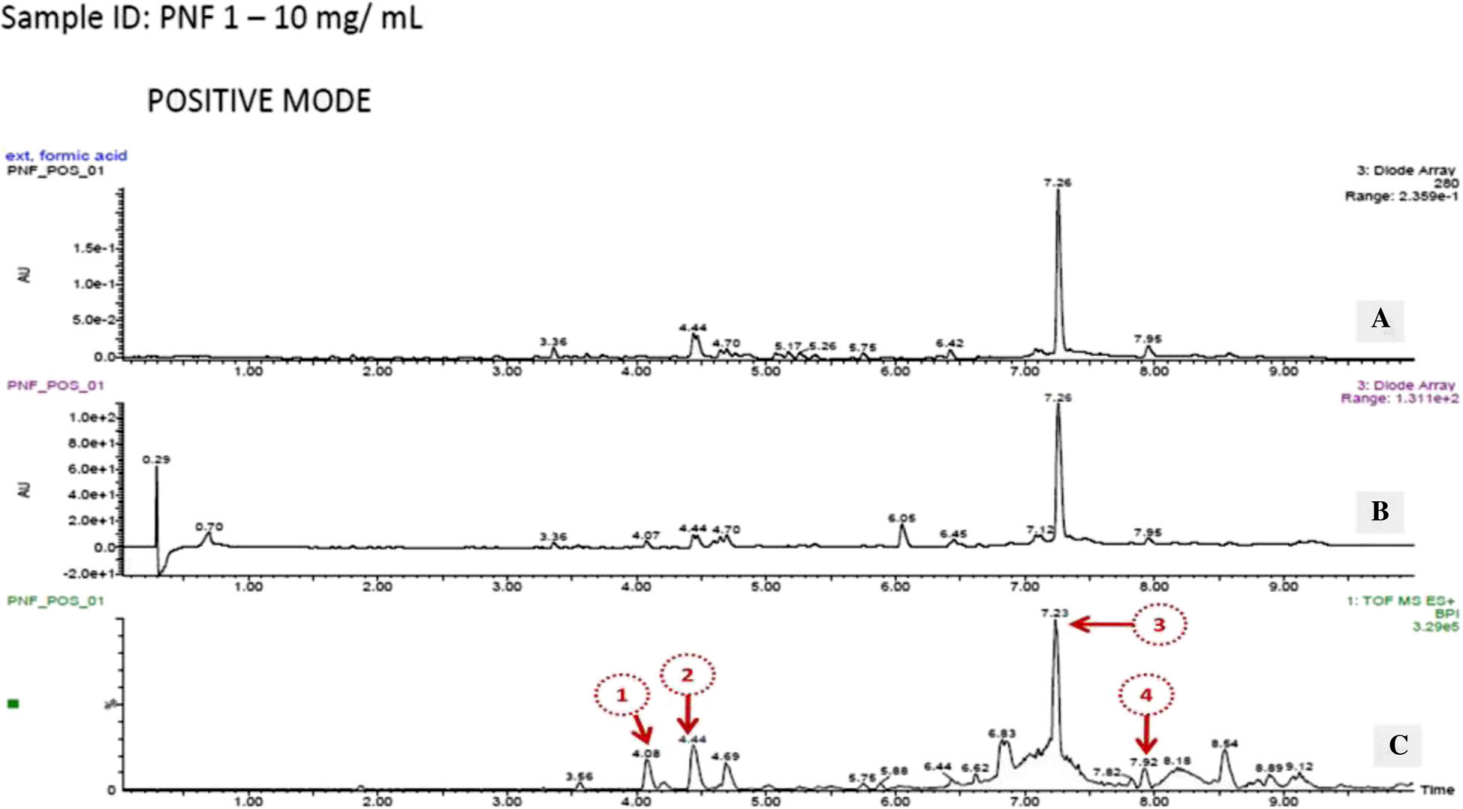
**LC-TOF/MS and UV diode array chromatograms of *P. niruri *fraction 1 (PNF1); (A) UV diode array spectra at 280 nm (B) UV diode array spectra at a range between 190 and 800 nm (C) TOF/MS peaks in the positive mode ionization.**

**Figure 5:**
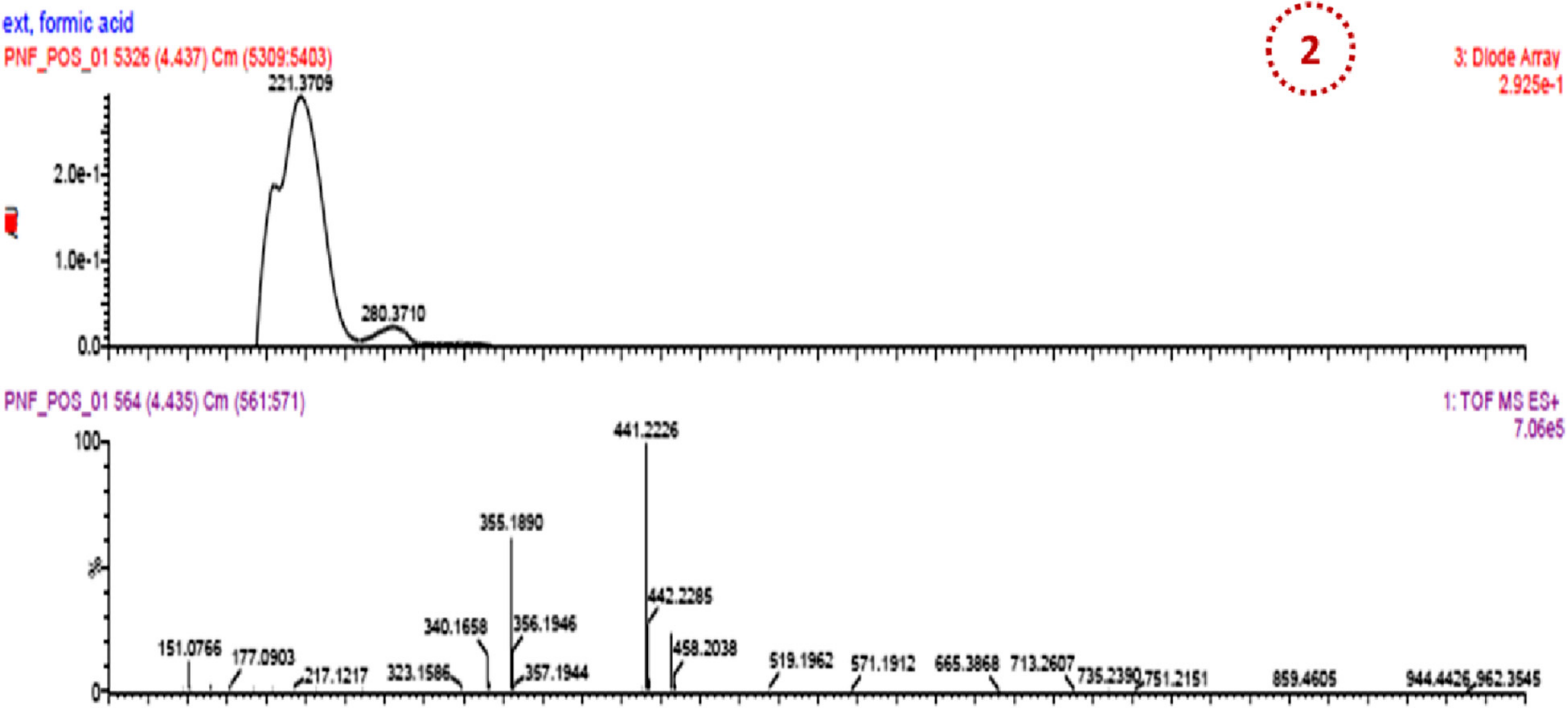
**UV max spectra and mass spectrum (TOF MS ES+) of peak no. 2 in *P. niruri *F1 (identified as 4-O-caffeoylquinic acid).**

**Figure 6:**
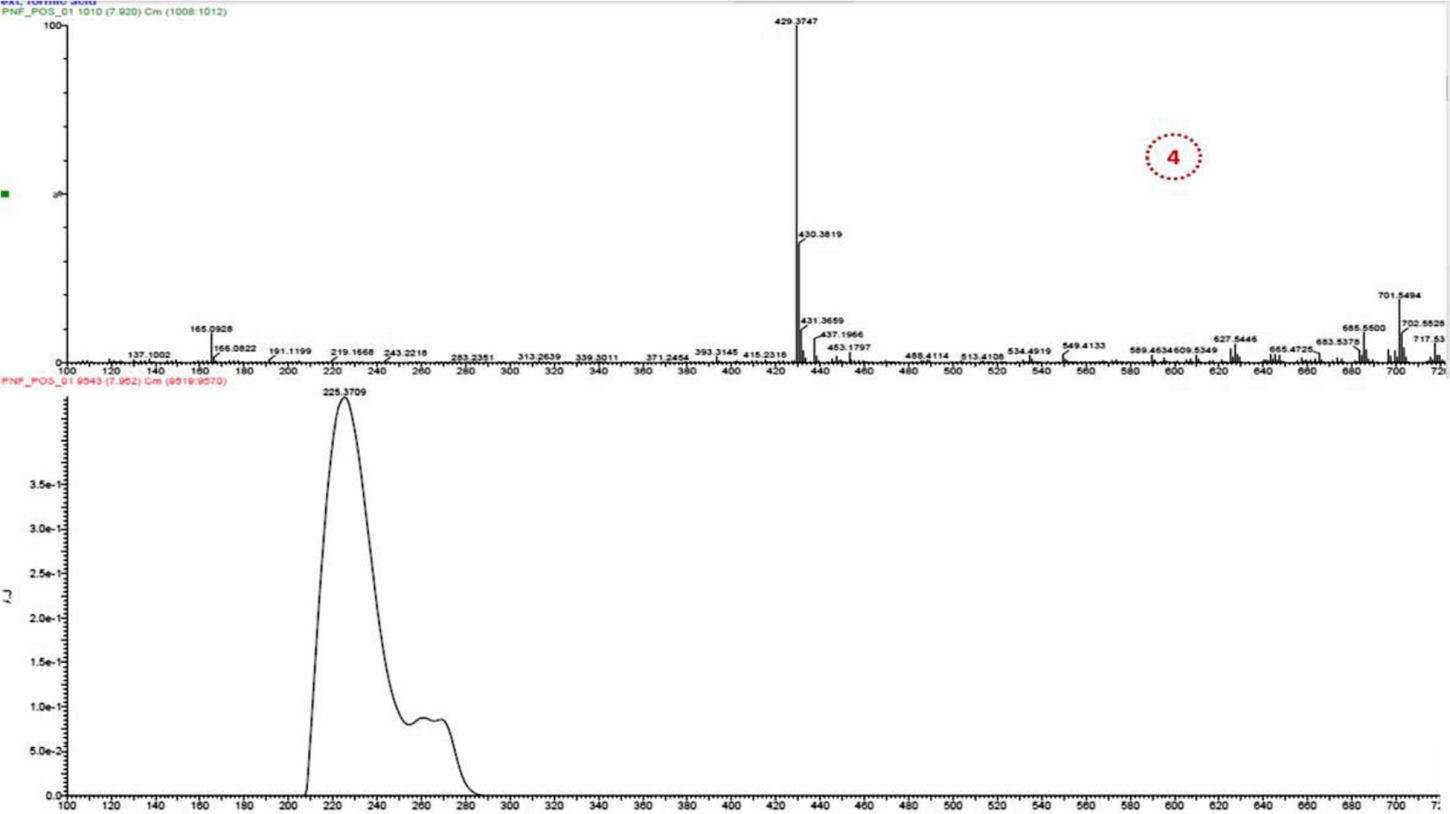
**UV max spectra and mass spectrum (TOF MS ES+) of peak no. 4 in *P. niruri *F1 (identified as quercetin 3-O-rhamnoside).**

LC/MS was performed on the PNF1 fraction, which exhibited higher activity to proliferate the PBMCs. Subsequently, by LC/MS/MS using the positive ionization mode, four peaks were observed from PNF1 (Figures 4, 5, 6 and Table 1). However, only peak numbers 2 and 4 were identified. Peak number 2 (RT = 4.454 min, λ = 221 and 280 nm, MW = 355) (Figure 5) had [M + H]^+^ at *m*/*z* 356 and was identified as caffeoylquinic acid (an isomer of chlorogenic acid) with fragments at *m*/*z* 340 (loss of CH_3_) and predominant fragments at *m*/*z* 191,165,151, and 147. Peak number 4 (Figure 6) (RT = 7.96 min, λ = 225 nm, MW = 448) had [M + H-H_2_O]^+^ at *m*/*z* 430 and was identified as quercetin 3-O-rhamnoside with the loss of H_2_O, and with the loss of rhamnoside, the ion appeared at *m*/*z* 303 and was identified as quercetin with other fragments at *m*/*z* 219, 205 and 165 [13,14].

**Table 1:** **Putative identification of main components of *P. niruri’*s active fraction**

**Plant fraction**	**Rt.time (min)**	**λ (nm)**	**Molecular weight**	**[M + H] *m*/*z***	**MS/MS fragmentation**	**Tentative identification**	**Molecular formula**
PNF1	4.437	221, 280	355	356	340, 191, 165 , 151, 147	4-O-Caffeolquinic acid	C_16_H_18_O_9_
	7.92	225	448	430	303, 219, 205, 165	Quercetin3-O-rhamnoside	C_21_H_20_O_11_

## Discussion

The objective of this study was to determine the roles of transforming growth factor β (TGFβ1), metalloproteinase-2 (MMP2), collagen αI (Collα1) and tissue inhibitor of metalloproteinase-1 (TIMP1) in preventing thioacetamide-induced liver cirrhosis in rats.

These results demonstrated that the mRNA expression levels of TGFβ1, Collα1, MMP2, and TIMP1 were unchanged in the control group; this supports the hypothesis that hepatic satellite cells (HSCs) were still in their quiescent state. However, these HSCs were activated by the presence of TAA and led to the high production of ECM and consequently high expression of TGFβ, Collα1, MMP2, and TIMP1.

PN treatment successfully prevented the high synthesis of ECM and reduced the mRNA expression of TGFβ, Collα1, and MMP2 compared with the TAA-treated group.

Most studies of human liver diseases and animal models of progressive fibrosis have demonstrated that TIMP1 mRNA expression was upregulated at early stages of fibrosis and because TIMP1 functions not only reduce MMP activity but also act on the suppression of apoptosis by HSCs [15]. In our findings, hepatic reduction in TIMP1 mRNA expression in the TAA-treated group can be explained as a consequence of increased HSC apoptosis [16].

**Figure 7:**
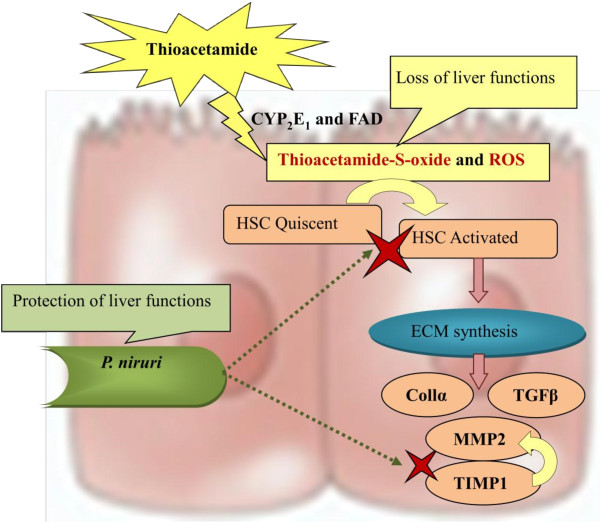
**Putative mechanism of *Phyllanthus niruri *hepatoprotective effect as a response to TAA-induced hepatotoxicity.**

Figure 7 shows the putative mechanism of the alteration of mRNA levels of the investigated genes in TAA-treated rats. First, TAA bioactivates into thioacetamide-S-oxide and other ROSs [17,18], and activates the HSCs, which, in turn releases more ECM and subsequently increases TGFβ gene expression that affects the release of collagen α and MMP1 and then TIMP1. Therefore, scar tissue develops, and the liver losses its normal functions, anatomical shape and architecture [19,20]. Treatment with PN significantly reduced the effect of thioacetamide toxicity as follows: 1) removing the causative stimuli of TAA, neutralizing ROSs by their high antioxidant content and attenuating endogenous antioxidant enzymes to their normal levels; 2) maintaining HSCs in their quiescent state; and 3) increasing the release of TIMP1 to counter balance MMP2 and complete remodeling of the hepatocyte cellular system that preserves or sustains normal liver function, shape, and appearance.

These findings confirm the previous findings of Wills and Asha [21] who suggested that the hepatoprotective role of *Lygodium flexuosum* plant extract is because of the reduced mRNA levels of growth factors, proinflammatory cytokines, and other signaling molecules, which are involved in hepatic fibrosis including TGFβ1, procollagen-I, and TIMP1. Additionally, Chen *et al*. [22] demonstrated the hepatoprotective effects of silymarin against TAA-induced liver damage to be caused by downregulation of hepatic MMP2, TIMP1,TGFβ1, COLα1 and other genes in the mouse model of chronic liver fibrosis. Although the antifibrotic and hepatoprotective properties of the silibinin–phosphatidylcholine-vitamin E complex in the rat model of liver fibrosis stimulated by bile duct ligation and dimethylnitrosamine administration have been postulated to cause reduced mRNA expression levels of procollagen type I, TGFβ1, TIMP1, and MMP2, the administration of the complex has also been reported to reduce hepatic stellate cell activation and proliferation with collagen deposition [23].

The isolated chemical constituents included in *P. niruri* (4-O-caffeoylquinic acid and quercetin 3-O-rhamnoside) can further interpret the abovementioned hepatoprotective activity. 4-O-Caffeoylquinic acid, which is classified as a tannin, has been isolated previously from *P. niruri* and proven to possess antioxidant, immunomodulatory, and hepatoprotective effects in several *in vivo* and *in vitro* assays [24-26]. However, quercetin 3-O-rhamnoside belongs to the flavonoid group of compounds that exhibits a wide range of pharmacological benefits including antimicrobial, antiviral, antioxidant, gastroprotective, hepatoprotective, anti-inflammatory and chemopreventive effects [27-30]. Moreover, potential working mechanisms of flavonoids during injuries and tissue damage include the following: the interference of ≥3 different free radical producing systems and an increase in function of the endogenous antioxidants CAT, SOD, and GPX [31]. Quercetin 3-O-rhamnoside has been confirmed in *Phyllanthus* species and other species of the Euphorbiaceae family [32-34]. Finally, both isolated chemical compounds from *P. niruri* (tannins and flavonoids) are classified as “polyphenols”, which are one of the most frequent and ever-present groups of plant metabolites, and have an important role in human and animal diets [35]. Recent studies have shown that plant-derived polyphenols are promising nutraceuticals for the control of various disorders, such as cardiovascular, neurological, and neoplastic diseases [36]. In addition, plant-derived polyphenolic compounds have hepatoprotective activity against different types of liver damage inducers, such as CCL_4_[37], paracetamol [38], and thioacetamide [39], which explains the high interest and initiation of many studies to evaluate the biological activity and bioavailabilities of polyphenolic compounds.

## Conclusion

This present study contributes significant knowledge to our understanding of the mechanism that underlies the hepatoprotective effect of *Phyllanthus niruri,* which is suggested to be through the regulation of TGFβ, Collα1, MMP2, and TIMP1 genes expression. The isolated chemical compounds (4-O-caffeoylquinic acid and quercetin 3-O-rhamnoside) of *Phyllanthus niruri* might have direct consequences for hepatoprotective activity. Therefore, promising approaches from this study must focus on TGFβ, Collα1, MMP2, and TIMP1 genes expression to develop new therapy for the treatment of liver cirrhosis.

## Competing interests

The authors declare that they have no competing interests.

## Authors’ contributions

Conceived and designed the experiments: ZA MS, performed the experiments: ZA MS, analyzed the data: ZA MS MK, contributed reagents/materials/analysis tools: MA HM ZA MS, wrote and revised the paper: ZA MA. All authors read and approved the final manuscript.

## Pre-publication history

The pre-publication history for this paper can be accessed here:

http://www.biomedcentral.com/1472-6882/13/160/prepub
